# Genomic insights into the prevalence and genetic diversity of *Salmonella* in chicken eggs in Saudi Arabia

**DOI:** 10.3389/fmicb.2026.1760213

**Published:** 2026-02-25

**Authors:** Amani T. Alsufyani, Rashed Bin Jaddua, Fahad M. Alreshoodi, Mohammed Alarawi, Sulaiman M. Alajel, Saleh Alaqeel, Elaf Alshdokhi, Ashwaq Alhamed, Hatim Almutairi, Khaloud O. Alzahrani, Norah Alotaibi, Abdullah Alajlan, Khalid S. Almaary, Turki M. Dawoud, Takashi Gojobori, Séamus Fanning, Ahmad M. Aljohani, Lenah E. Mukhtar

**Affiliations:** 1 Saudi Food and Drug Authority (SFDA), Riyadh, Saudi Arabia; 2 Department of Botany and Microbiology, College of Science, King Saud University, Riyadh, Saudi Arabia; 3 UCD-Centre for Food Safety, University College Dublin, Belfield, Dublin, Ireland; 4 Biological and Environmental Sciences and Engineering (BESE) Division, King Abdullah University of Science and Technology, Thuwal, Saudi Arabia; 5 Computer, Electrical and Mathematical Sciences and Engineering Division, King Abdullah University of Science and Technology, Thuwal, Saudi Arabia; 6 Institute for Global Food Security, The Queen’s University of Belfast, Belfast, Northern Ireland, United Kingdom

**Keywords:** antibiotics, antimicrobial susceptibility, chicken eggs, genomics, *Salmonella*, salmonellosis, serovar

## Abstract

**Introduction:**

*Salmonella* enterica remains one of the leading causes of foodborne illness globally, with poultry and eggs recognized as major transmission vehicles. In Saudi Arabia, data integrating genomic epidemiology with conventional microbiological surveillance in eggs remain limited. This study aimed to provide the first comprehensive genomic characterization of *Salmonella* isolated from chicken eggs in the Kingdom, assessing prevalence, serovar distribution, virulence determinants, and antimicrobial resistance (AMR) profiles to better inform food safety risk assessment within a One Health framework.

**Methods:**

A total of 260 chicken eggs were collected and analyzed using conventional microbiological culture methods for Salmonella detection. Confirmed isolates underwent whole-genome sequencing (WGS) to determine serovar identity, virulence-associated genes, and antimicrobial resistance determinants. Comparative genomic analyses were conducted to evaluate differences between isolates recovered from eggshell surfaces and egg contents, with particular focus on multidrug resistance (MDR) genes, plasmid-mediated *β*-lactamases, and fluoroquinolone resistance determinants.

**Results:**

*Salmonella* was detected in 9% of the analyzed samples. Contamination was more frequently identified on eggshells than within egg contents, suggesting a substantial contribution of environmental exposure during handling and distribution. Twelve distinct serovars were identified, with *Salmonella Enteritidis* and *Salmonella typhimurium* predominating.Eggshell isolates demonstrated broader serovar diversity but carried fewer antimicrobial resistance determinants. In contrast, isolates recovered from egg contents were associated with clinically significant serovars and exhibited a higher prevalence of antimicrobial resistance genes. Whole-genome analysis revealed multidrug resistance profiles, including plasmid-mediated *β*-lactamase genes and fluoroquinolone resistance determinants, indicating the potential for dissemination of resistant strains through the food chain.

**Discussion:**

The findings demonstrate dual contamination pathways in eggs: external contamination linked to environmental handling factors and internal contamination associated with serovars of greater clinical relevance and elevated AMR gene burden. The detection of multidrug resistance determinants highlights the risk of horizontal transmission of resistance elements across foodborne bacteria.These results underscore the importance of integrating genomic surveillance into national food safety systems to strengthen risk assessment, guide targeted control measures, and support antimicrobial resistance monitoring. Within the Saudi context, implementing WGS-based surveillance aligns with One Health principles and enhances preparedness against emerging foodborne AMR threats.

## Introduction

Chicken eggs are considered a primary source of food and are often implicated in the transmission of *Salmonella,* making them a major source of foodborne illness, i.e., salmonellosis (Shaji et al., 2023). Indeed, egg-borne salmonellosis is a common worldwide public health concern that has been documented with major health and economic implications (Pal et al., 2020). Annually, the global economic burden of salmonellosis has been estimated to exceed €2.8 billion (Shekhar, 2018). Salmonellosis (*Salmonella* infection) affects millions of individuals globally annually, and in the United States (US) alone, it accounts for around 11% of all foodborne infections, with around 35% of those cases requiring hospitalization and about 28% developing complications that may lead to death (Billah and Rahman, 2024; Scallan et al., 2011). Recent outbreaks continue to highlight the relevance of eggs in *Salmonella* transmission. For instance, in 2024, a multi-state outbreak in the US linked to contaminated eggs resulted in 93 confirmed cases across 12 states, with 34 individuals hospitalized (CDC, 2025). Similarly, the European Union (EU) consistently reports eggs and egg products as among human salmonellosis (Sarno et al., 2021).

Despite the well-known public health significance of salmonellosis, recent evidence indicates that extensive national data about the prevalence, epidemiology, and transmission sources of *Salmonella* in Saudi Arabia are still limited, highlighting a critical knowledge gap (Alhumaidan, 2024). In Saudi Arabia, *Salmonella* spp. are frequently identified as significant bacterial agents linked to foodborne illnesses, resulting in a considerable number of reported cases annually (Alhumaidan, 2024). National surveillance data reveal variable incidence rates in recent years, indicating persistent exposure hazards and the possible underestimating of the actual disease burden due to underreporting and constraints in routine source attribution. In Saudi Arabia, although salmonellosis is typically documented as isolated incidents rather than persistent outbreaks, the particular food sources responsible for human infections are inadequately defined (Alhumaidan, 2024; Alhumaid et al., 2024). In the Gulf Cooperation Council (GCC) region, encompassing Saudi Arabia, salmonellosis has been linked to contaminated food products of animal origin, such as chicken, eggs, dairy, and fresh produce (Mohamed et al., 2024). In addition, local epidemiological research have revealed *S. enteritidis* and *S. typhimurium* as the most commonly detected serovars in human infections, highlighting their significance for public health surveillance (Alhumaidan, 2024). Thus, comprehending the circulating serovars, antibiotic resistance patterns, and critical virulence factors is vital for enhancing preventative tactics, directing clinical management, and shaping food safety policies (Mohamed et al., 2024; Khan et al., 2024). *Salmonella* species (spp.) are gram-negative, facultative anaerobic, non-spore-forming flagellated bacilli belonging to the Enterobacteriaceae family (Lamas et al., 2018). *Salmonella* comprises more than 2,500 serovars, and *Salmonella enterica* subspecies enterica is the most prevalent serovar among them, which is responsible for approximately 99% of human *Salmonella* infections (Lamas et al., 2018).

Salmonellosis manifests with gastrointestinal symptoms like diarrhea, abdominal pains, nausea, vomiting, and fever, often within a timeframe of 12–72 h after the ingestion of contaminated food (Giannella, 1996). While Salmonellosis often resolves on its own without treatment within a period of 3–7 days, severe illness can occur in vulnerable populations, including children under the age of 5 or elderly people of age 65 and beyond, and individuals with compromised immune systems due to specific medical illnesses, such as, diabetes, liver or kidney disease, and cancer (Lamichhane et al., 2024; CDC, 2023; Bula-Rudas et al., 2015).

Accurate identification and typing of *Salmonella* strains are essential for outbreak tracking and public health response. The Centers for Disease Control and Prevention CDC and other global health agencies emphasize the importance of whole-genome sequencing (WGS) technology to facilitate strain-level differentiation, detect antimicrobial resistance (AMR) genes, enhance surveillance capabilities, and better control for salmonellosis incidence and outbreaks (CDC, 2024a; Struelens and Brisse, 2013).

Salmonellosis antibiotic therapy is recommended for severe cases and at-risk individuals, especially for cases with severe illness and a weakened immune system (Chen et al., 2013). Traditionally, *β*-Lactams (including penicillin derivatives, cephalosporins, and carbapenems), monobactams, and fluoroquinolones are the commonly used antibiotics to treat Salmonellosis (Lamichhane et al., 2024; Chen et al., 2013; Bhandari et al., 2023). However, the therapeutic efficacy of these drugs is increasingly threatened by the emergence of AMR. This rise in the AMR is often attributed to the extensive or unregulated use of antibiotics in animal husbandry, including poultry production (Abou-Jaoudeh et al., 2024).

AMR in poultry production represents a growing risk due to its significance for food safety, public health, and the spread of resistant bacteria across the food chain. In Saudi Arabia, government initiatives to address AMR are directed by a One Health framework that unifies the sectors of human, animal, and agrifood health. The Saudi Food and Drug Authority (SFDA) plays a central role in regulating antimicrobial usage and enhancing food safety monitoring in accordance with the Global Action Plan on Antimicrobial Resistance.

Saudi Arabia has implemented a National Action Plan on antibiotic Resistance (NAP-AMR 2022–2025), focusing on strengthening surveillance systems, improving antibiotic utilization in human and animal health, and enhancing laboratory capabilities for AMR detection (Kingdom of Saudi Arabia, 2026). The NAP-AMR emphasizes the significance of sentinel surveillance locations and laboratory-based monitoring for priority foodborne pathogens, such as *Salmonella*, in both human and animal sectors. Within this framework, the National Food Antimicrobial Resistance Surveillance System (NARSS), coordinated by the SFDA, has been recently established as a centralized platform to support systematic monitoring of antimicrobial resistance patterns and to facilitate evidence-based risk assessment and policy development. NARSS operates as a confidential, non-public platform, however it is essential for guiding national policy and regulatory decisions concerning food safety and antimicrobial resistance.

Despite these advancements, there is a notable lack of comprehensive genetic data on antimicrobial resistance determinants in foodborne pathogens, especially *Salmonella* linked to poultry eggs. The spread of AMR in *Salmonella* is frequently mediated by mobile genetic elements such as plasmids, integrons, and transposons, which contribute to the horizontal transfer of resistance genes across bacterial populations (Algarni et al., 2022). As a result, multidrug-resistant (MDR) *Salmonella* strains have become a growing concern, not only because they limit therapeutic options but also due to their association with increased morbidity, mortality, and healthcare costs (Algarni et al., 2022). For example, in the United States, an estimated 2 million people are affected annually by antibiotic-resistant bacteria, including *Salmonella*, leading to over 23,000 deaths (Slayton et al., 2015). This global increase underscores the urgent need for robust surveillance systems and tighter regulations on antimicrobial use in food-producing animals.

On the other hand, in Saudi Arabia, the MDR *Salmonella* is also a cause of concern due to the rapid growth of the egg production sector (Saudi Arabia, 2021). According to the Poultry and Products Annual Report 2021, Saudi Arabia produces approximately 5 billion eggs yearly, and exports over 5 billion eggs annually to neighboring GCC countries and recently, it reached self-sufficiency levels exceeding 100% (Saudi Arabia, 2021). However, studies on *Salmonella* in eggs in Saudi Arabia remain limited, regardless of their critical importance to the egg production industry. Most existing research in Saudi Arabia has focused on the prevalence of *Salmonella* in meat, vegetables, milk, and seafood, leaving a critical gap in understanding its presence in eggs and egg products (Moussa et al., 2010; al-Nakhli et al., 1999). Therefore, this study aims to fill that gap by assessing the prevalence, serovar distribution, antimicrobial resistance, and genomic diversity of *Salmonella* isolated from chicken eggs in Saudi Arabia using WGS technology.

This study is the first of its kind to focus on *Salmonella* from chicken eggs in Saudi Arabia, providing comprehensive genomic analysis and valuable insights into its prevalence and genetic diversity. These critical data contribute to both national surveillance and international public health efforts.

## Materials and methods

### Sample size and collection

Between April 2021 and April 2022, chicken egg samples were systematically collected from retail markets, supermarkets, and local groceries across various districts in Riyadh City, Saudi Arabia. Each sampled unit consisted of an egg carton containing 30 eggs. Cartoons were then transported immediately under ambient temperature to the Reference Laboratory for Microbiology at the Saudi Food and Drug Authority (RLM-SFDA). Upon arrival, all samples were visually examined to ensure eggshell integrity, and the eggs to be tested were intact. Two random eggs from each pack were selected as a single analytical sample (*n* = 260 duplicates). The egg samples were obtained from major poultry companies in Saudi Arabia that supply and distribute eggs across Saudi cities.

### Recovery, isolation, and identification of *Salmonella*

**Figure 1:**
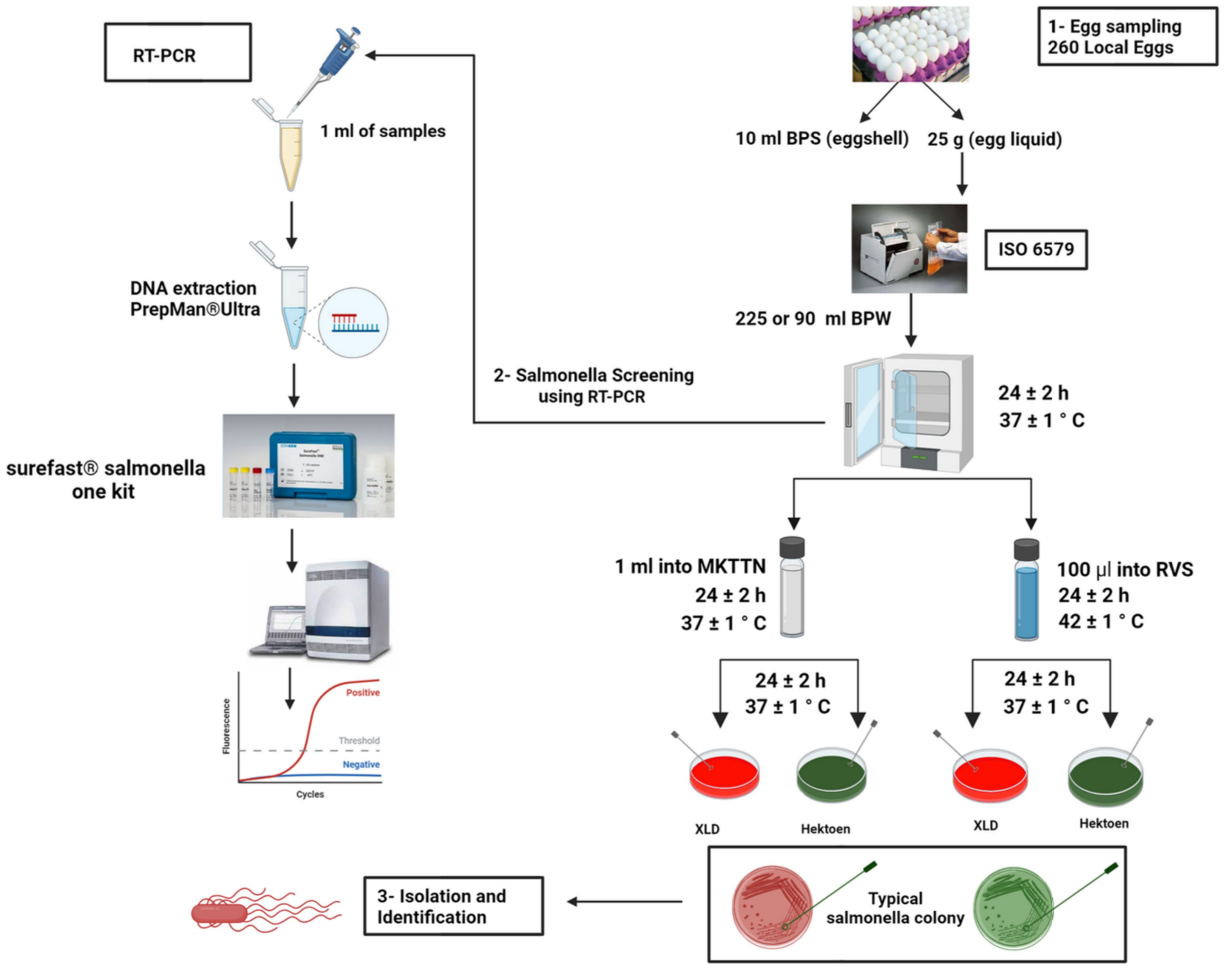
Workflow describes the recovery and identification of *Salmonella* from chicken egg samples using the rinsate method. The procedure adheres to ISO 6579-1:2017 standards for both initial detection and confirmation. Positive samples were further serotyped according to the same standard protocol. This figure was created with BioRender.com.

Eggshells and contents of each sample were then processed and tested separately for the detection of *Salmonella*. First, *Salmonella* was recovered according to the shell rinse method (Musgrove et al., 2005). Each intact egg was collected and placed in a sterile stomacher bag (Stomacher^®^, Seward, United Kingdom) and rinsed with 10 mL phosphate-buffered saline 10X (PBS) (Invitrogen™, Thermo Fisher Scientific United States) for 1 min while gently massaging the shell through the bag to detach surface bacteria. Then the rinsates were stored at 4 °C for 24 h (hrs), then combined with 90 mL of buffered peptone water (BPW) (Oxoid; Hampshire; England) and incubated at 37 °C for 24 ± 2 h. For internal egg content analysis, the surface of the eggshell was sterilized by immersion in 70% ethanol for 2 min, air-dried, and then cracked aseptically using a sterile knife into a sterilized stomacher bag. Then, about 25 mL of the egg content was thoroughly mixed with 225 mL of BPW and homogenized for 2 min using a stomacher (AES Smasher, BioMerieux, France), followed by incubation at 37 °C for 24 ± 2 h. Following the recovery of *Salmonella*, the standard method described by the International Organization for Standardization was used to isolate the Salmonella ISO 6579-1:2017 (ISO, 2024). Briefly, after the pre-enrichment, samples underwent selective enrichment incubation using two media broths: (1) Muller-Kauffmann tetrathionate-novobiocin broth (MKTTn broth; Oxoid; Hampshire; England), and (2) Rappaport-Vassiliadis medium with soya (RVS broth; Condalab; Madrid; Spain) at 37 °C for 24 ± 2 h and 42 °C for 24 ± 2 h, respectively. Aliquots of the cultures were then streaked onto Xylose Lysine Deoxycholate (XLD; Oxoid™, England), and (Hektoen; Condalab, Spain) selective agar plates and incubated at 37 ± 1 °C for 24 ± 2 h. About five resumptive colonies with suspected *Salmonella* morphology were selected from selective media and sub-cultured at 37 °C for 24 h into Nutrient Agar (NA; Oxoid, England) plates for purification. After incubating the nutrient agar plates at 37 °C for 24 h. Typical *Salmonella* colonies were collected and confirmed by Real-Time PCR (Applied Biosystems, United States) using SureFast *Salmonella* ONE real-time PCR kit (SureFast^®^ Salmonella ONE, CONGEN, Germany) following the manufactured instructions. After that, the confirmed *Salmonella* isolates were serotyped by slide agglutination targeting somatic (O) and flagellar (H) antigens in accordance with the White-Kauffmann-Le Minor scheme (Guibourdenche et al., 2010). *Salmonella Typhimurium* ATCC 14028 and *Salmonella Enteritidis* ATCC 13076 were used as the positive control strains. Lastly, any pure isolates were stored in 70% BPW and 15% glycerol and 15% distilled water as stock cultures at −80 °C in the biobank at RLM-SFDA (Figure 1). Serovar assessment was obtained by slide agglutination and were subsequently verified by *in silico* serotyping based on whole-genome sequencing.

### Antimicrobial susceptibility testing

Antimicrobial susceptibility test (AST) was applied using the broth microdilution method with Sensititre™ TREK diagnostic system commercial panels (NARMS CMV3AGNF, EUVSEC3, and GNX3F; Thermo Fisher Scientific, United States) for all 23 confirmed *Salmonella* isolates. The broth microdilution method implemented using the Sensititre™ system is consistent with Clinical and Laboratory Standards Institute (CLSI) reference methodology for AST, as described in CLSI M07, and is widely accepted for determining MIC values for Enterobacterales, including *Salmonella* (CLSI M07, 2026). These panels were selected to cover a broad range of antibiotics commonly used in both human medicine and animal production to provide resistance profiling. Inoculation and incubation were performed according to the manufacturer’s guidelines. With a quality control strain, *Escherichia coli* ATCC 25922, and *Pseudomonas aeruginosa* ATCC 27853 used throughout the testing. The Sensititre panel plates comprised a total of 32 antibiotics from 12 different classes. After that, the extended-spectrum *β*-lactamase (ESBL) production was assessed using the EUVSEC2 panel, following CLSI interpretive criteria (Clinical and Laboratory Standards Institute, 2024). Minimum inhibitory concentrations (MICs) of each antibiotic were interpreted according to CLSI antimicrobial susceptibility testing standards (CLSI M100 Ed. 33), which provide internationally recognized clinical breakpoints for Enterobacterales, including *Salmonella*, and are widely used for clinical and surveillance purposes (Clinical and Laboratory Standards Institute, 2024). CLSI criteria were applied for antimicrobial agents with established interpretive breakpoints to ensure consistency and comparability with global antimicrobial resistance surveillance studies (Clinical and Laboratory Standards Institute, 2024).

As there are no CLSI interpretive breakpoints for streptomycin, azithromycin, cefoxitin, and ceftiofur for *Salmonella*, MIC interpretive standards were followed based on the National Antimicrobial Resistance Monitoring System (NARMS) (CDC, 2024b), NARMS criteria were selected because they are specifically designed for monitoring antimicrobial resistance in foodborne *Salmonella* and are commonly used in food and animal surveillance programs. The antibiotic tigecycline was interpreted according to the USA-Food and Drug Administration (US-FDA) guidelines (Research C for DE and, 2024).

### DNA extraction and whole-genome sequencing

Genomic DNA was extracted from pure colonies using the DNeasy PowerSoil Pro Kit (Qiagen, Germany) following the manufacturer’s instructions. DNA purity was confirmed by the A260/A280 ratio (target ≥1.8) using QIAxpert spectrophotometer (Qiagen, Germany). DNA concentrations were quantified using QFX Fluorometer (Denovix Inc., Delaware, United States) according to the manufacturer’s directions. DNA libraries were prepared using QIAseq FX DNA Library Kit (Qiagen, Germany). The samples were then normalized and quantified by Qubit quantification and pooled to generate a 4 nM library. Paired-end sequencing was performed on the Illumina NovaSeq platform, using 2 × 150 cycles with 5% PhiX control (Illumina Inc.).

### Bioinformatics analyses

Quality-controlled Illumina paired-end reads were screened with FastQC v0.12.1,^1^ adapters and low-quality tails were removed with Trimmomatic v0.39.^2^ Only read pairs that passed post-trimming quality filtering (default parameters) were retained. De-novo assembly was performed with the SPAdes v3.15.2^3^ in normal mode; contigs shorter than 500 bp were discarded. Assembly quality was evaluated with QUAST v5.0.2.^4^

Assembled genomes were used to assign sequence types with MLST 2.19.0^5^ and to detect antimicrobial-resistance genes with ResFinder 4.1^6^ under default cut-offs. Plasmid replicons were identified with Abricate v1.0.1 (via the TORMES v1.3.0 pipeline) against the PlasmidFinder database (accessed on 28 November 2023)^7^; default Abricate settings were applied, but only matches with ≥90% identity and ≥ 90% coverage were retained, and replicon names follow PlasmidFinder nomenclature. Association of antimicrobial-resistance genes (ARGs) with plasmids was inferred whenever an ARG and a plasmid replicon co-localized on the same contig after applying the ≥90% identity and ≥90% coverage filters. Virulence factors were annotated against VFDB (accessed on 28 November 2023)^8^ under more-stringent cut-offs of ≥ 90% identity and ≥50% coverage. Unless explicitly stated, all tools were run with their remaining default parameters.

### Statistical analysis and data visualization

**Figure 2:**
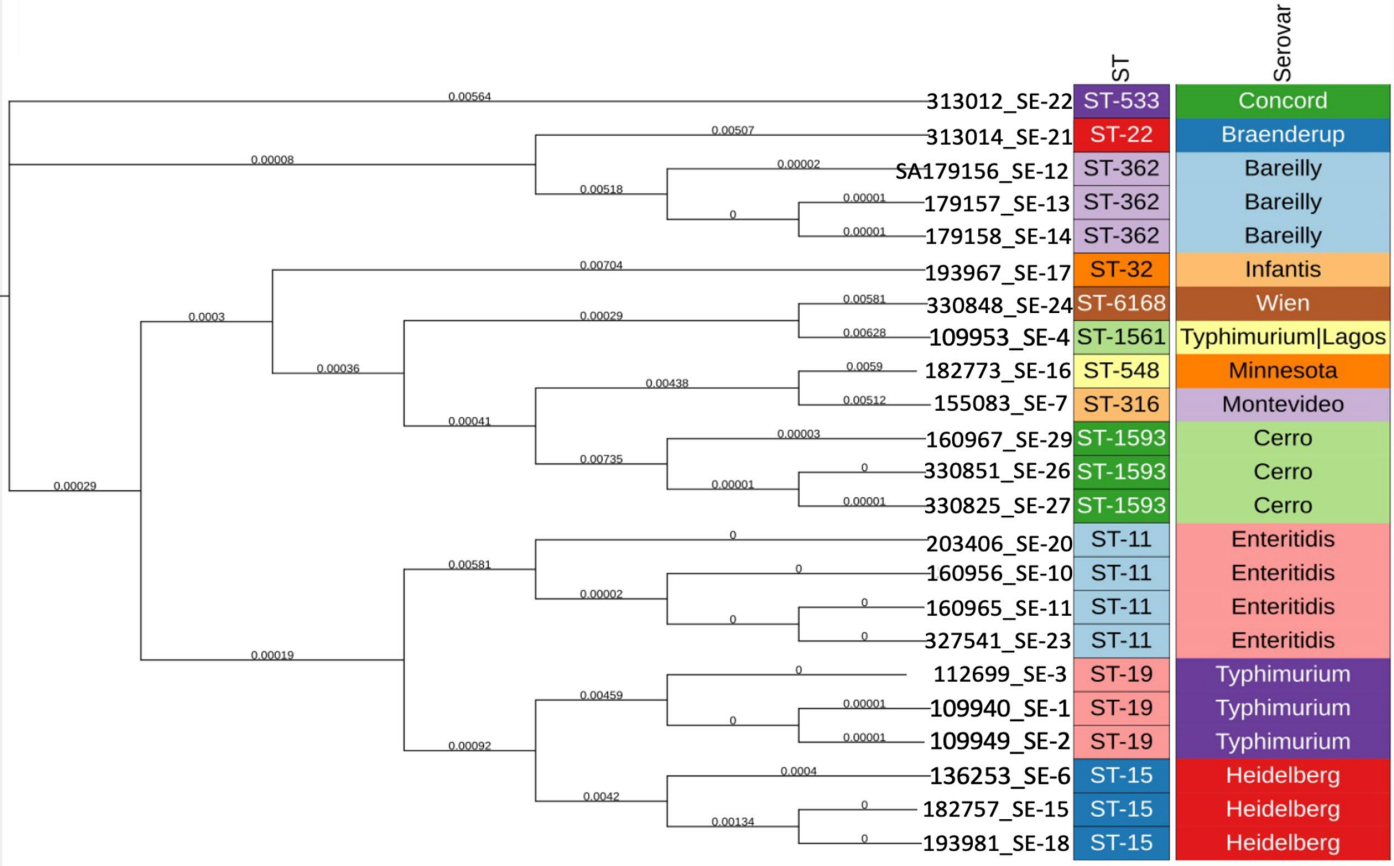
Phylogenetic tree showing genetic relatedness of *Salmonella* isolates based on sequence typing (ST) distances. Branch lengths reflect the degree of allelic variation among isolates.

**Figure 3:**
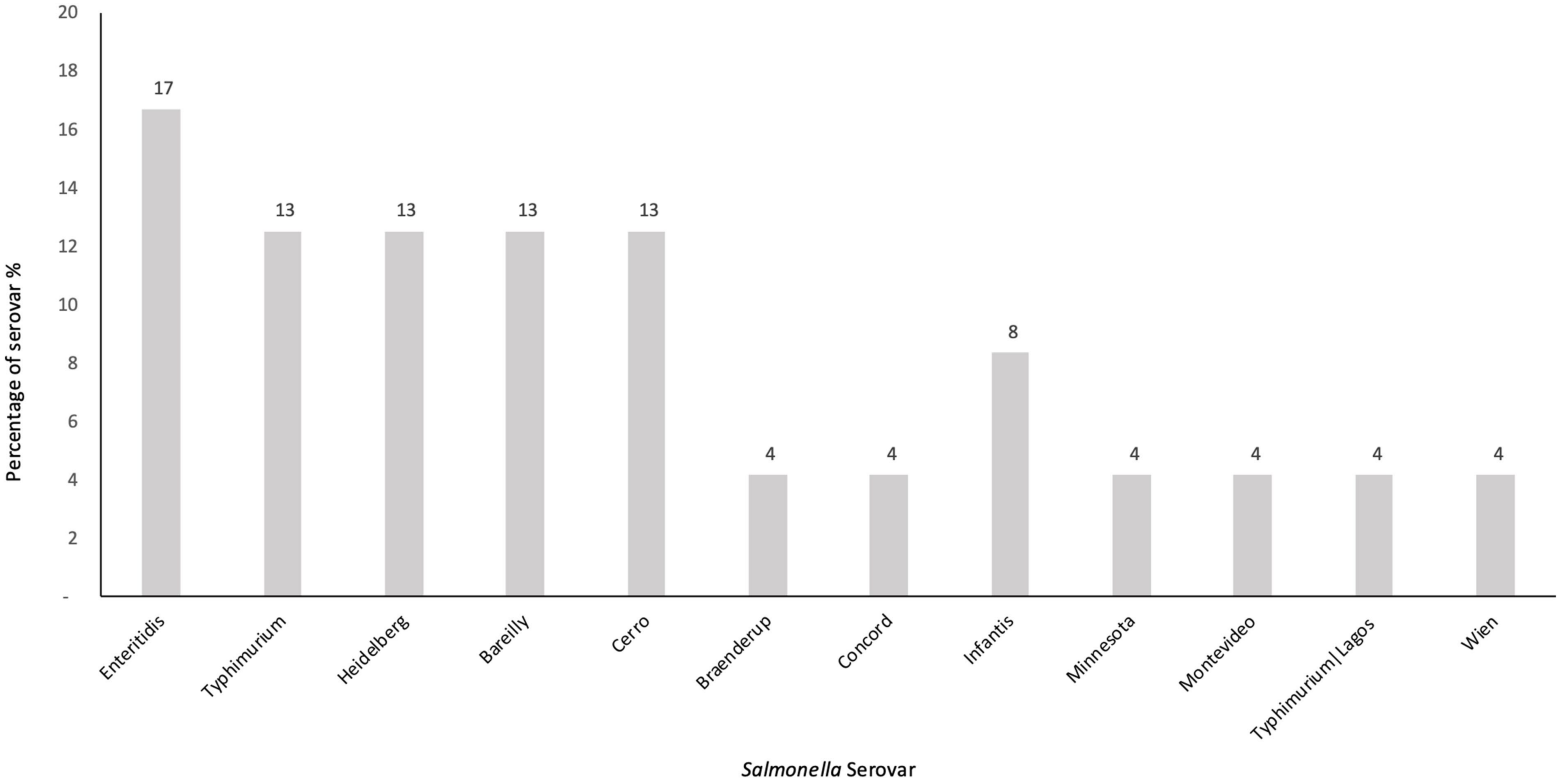
The figure illustrates the percentage of each *Salmonella* serovar detected in egg samples.

**Figure 4:**
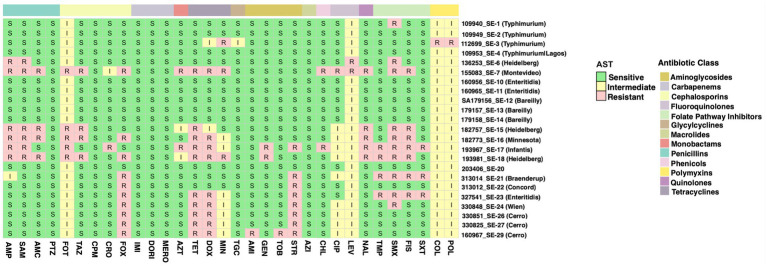
Antibiotic susceptibility of the *Salmonella* serovars to a total of 32 antibiotics from 12 distinct classes shown as a colored. Susceptibility categories were interpreted according to the Clinical and Laboratory Standards Institute (CLSI) criteria. The letter R with red color refers resistant, I with yellow color is intermediate, and S with green color is sensitive.

**Figure 5:**
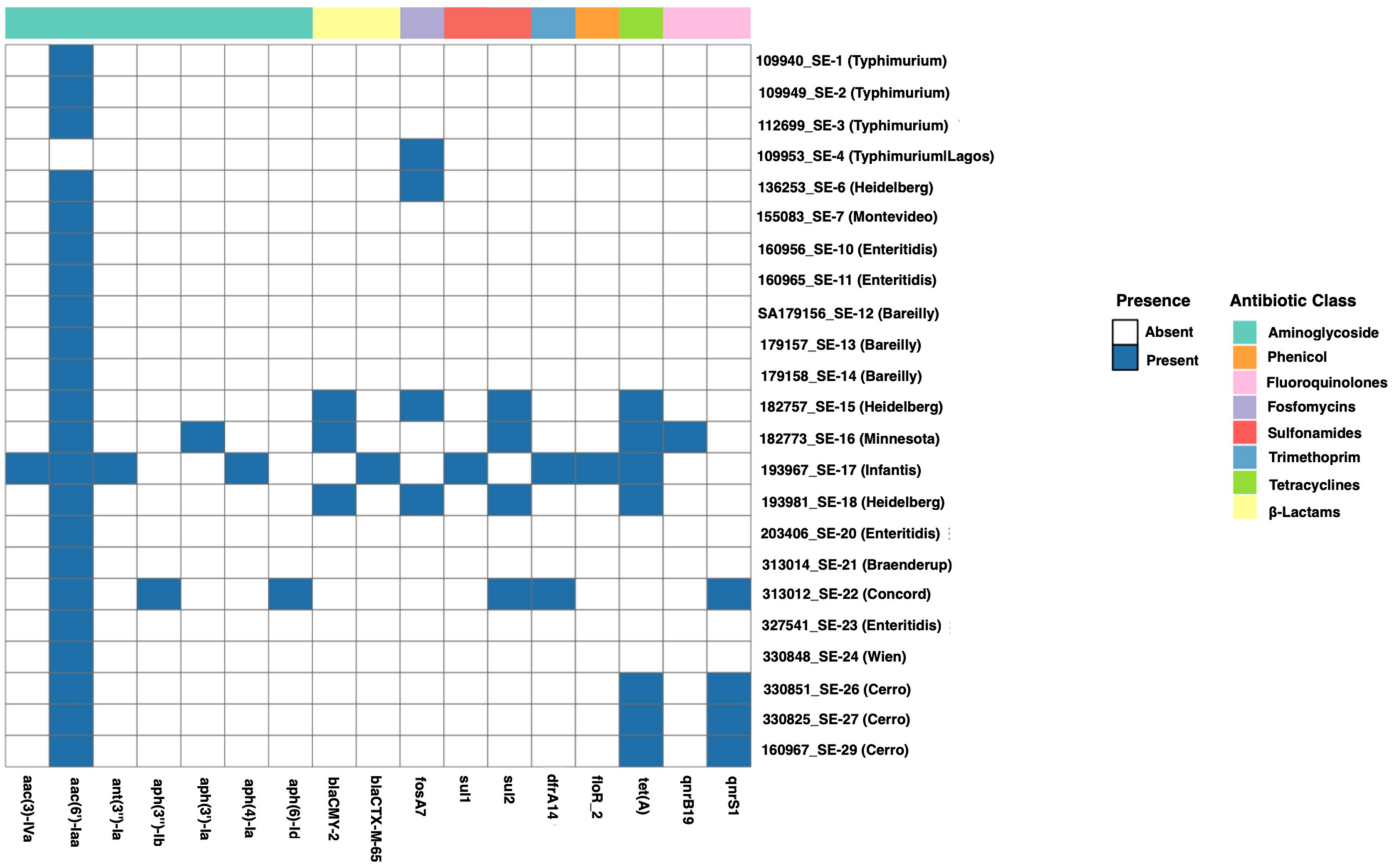
Heatmap showing the *Salmonella* serovars’ antimicrobial resistance genes presence and absent. Each tile represents the presence (blue) or absence (white) of genes. Genes are grouped by antimicrobial class, shown as a colored.

**Figure 6:**
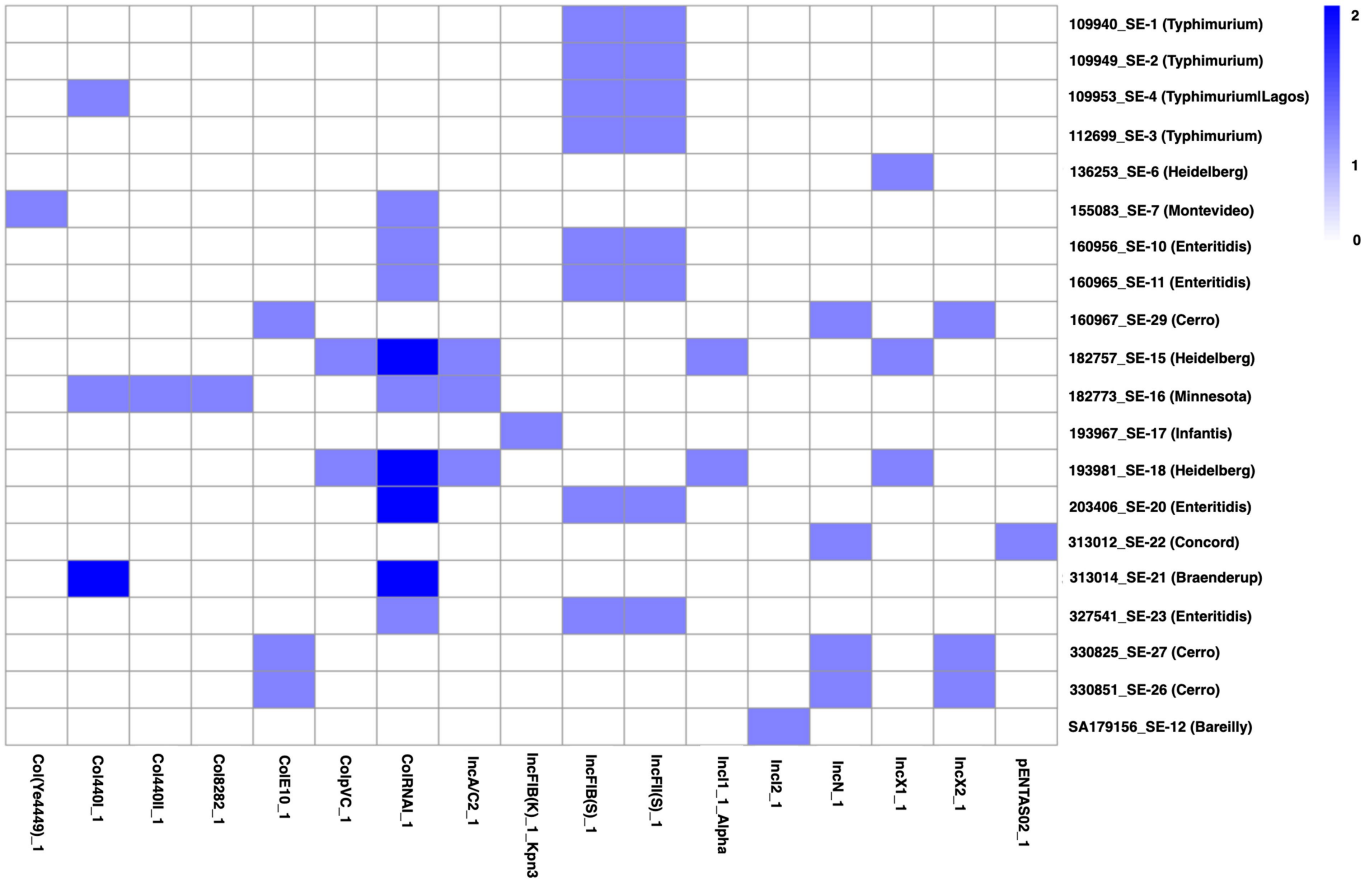
Heatmap showing the distribution of *Salmonella* serovars’ plasmid replicons. The color scale shows the number of genes present per isolate, from 0 (white none), 1 (light blue, one gene) to 2 (dark blue, two genes).

**Figure 7:**
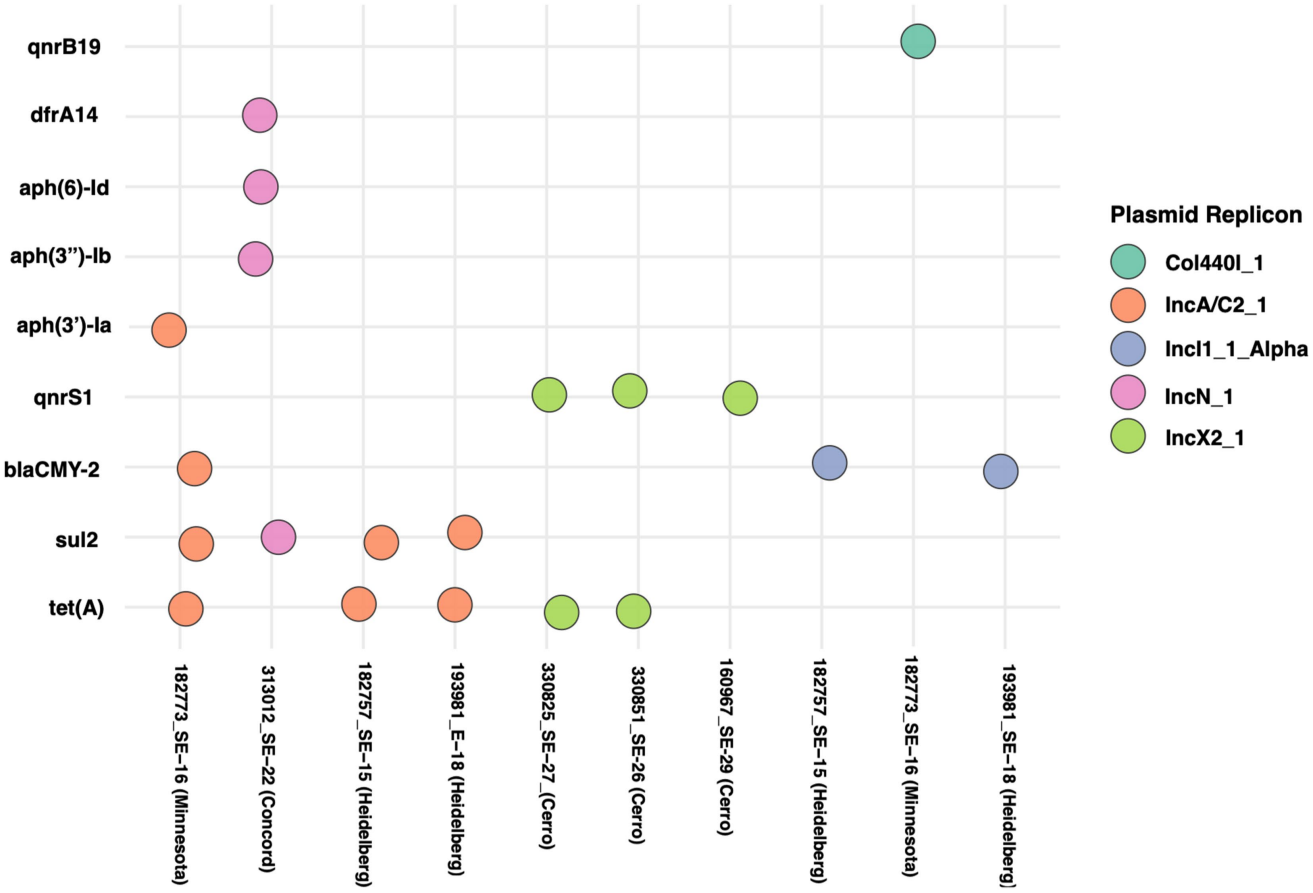
Distribution of antimicrobial resistance (AMR) genes across samples and their associated plasmid replicons. Each column represents a sample ID and each row represents an AMR gene. Colored bubbles indicate the presence of a specific plasmid replicon carrying that gene in the corresponding sample. Bubble colors distinguish plasmid replicon.

**Figure 8:**
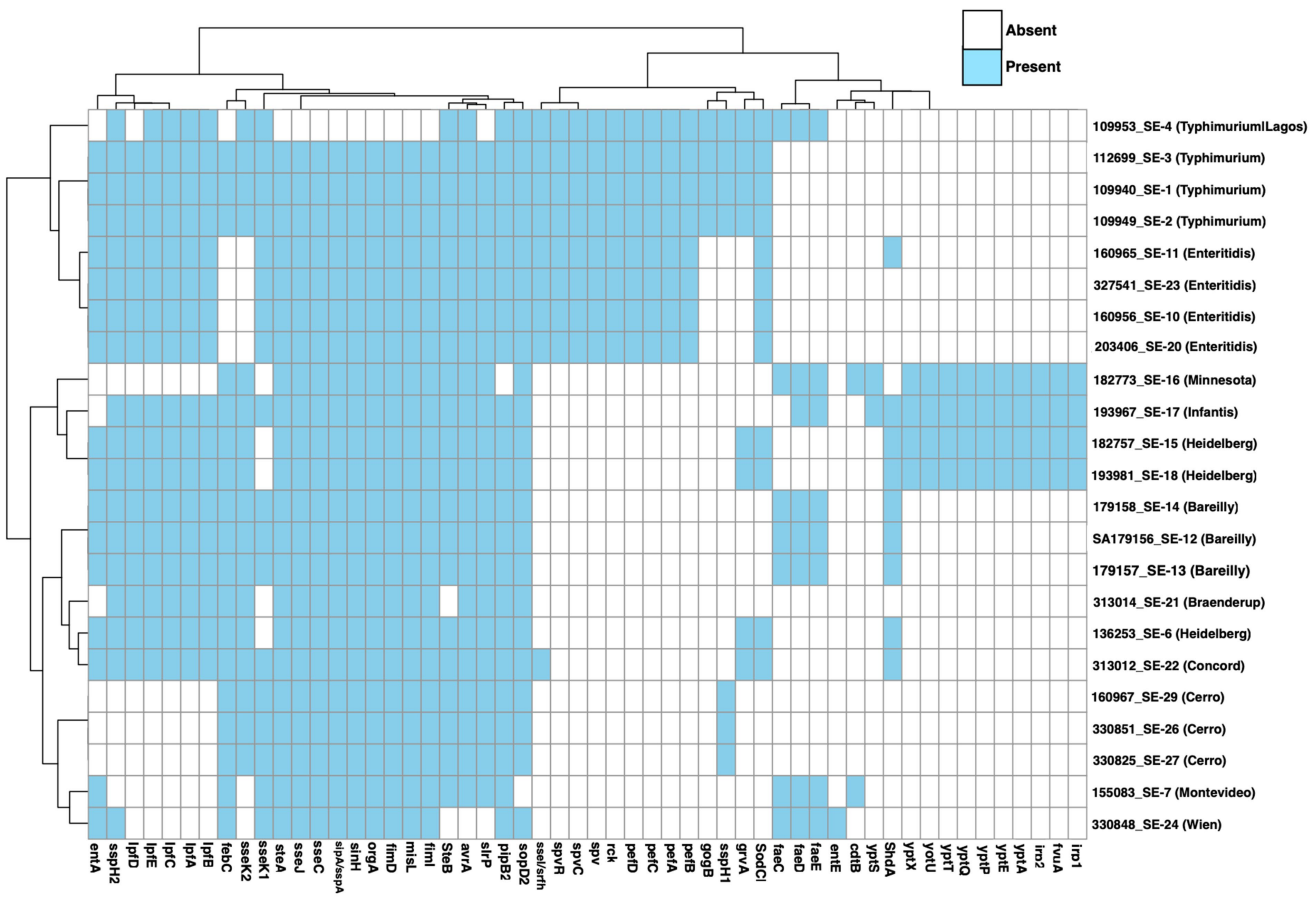
Heatmap showing *Salmonella* serovars’ virulence genes presence and absent. Rows reflect individual isolates. Each tile represents the presence (blue) or absence (white) of genes.

**Figure 9:**
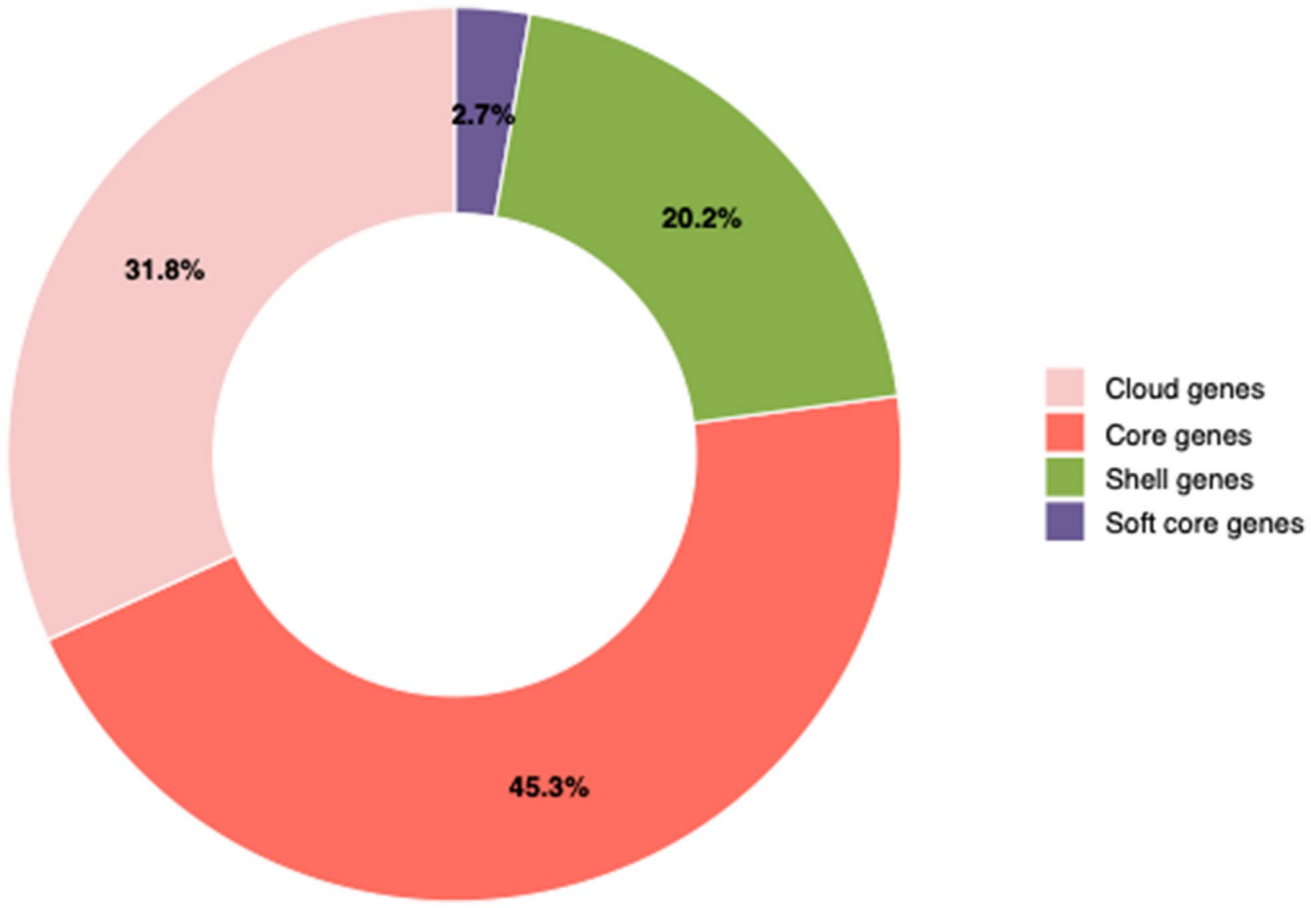
Distribution of genes in the *Salmonella* pangenome. The analysis showed percentages of core genes, cloud genes, shell genes, and soft-core genes.

All figures and data visualizations were generated using R software. From CSV files prepared after bioinformatics processing, except for Figure 2 (Core Team, 2022). The complete R scripts used for AMR profiling, ARGs, plasmid–ARG associations, virulence gene heatmaps, and pangenome visualization (Figures 3–9) are openly available in our GitHub repository at: https://github.com/Amanitaa89/WGS-*Salmonella*-Eggs-Scripts. All R scripts were subsequently verified in a clean R environment, with outputs cross-checked against the raw datasets and figures. Figure 2 was generated using IQ-TREE v2.2.2.7 from a core genome alignment produced by Panaroo v1.5.0 and visualized with iTOL. The overall workflow illustration was created using BioRender.com (Scientific Image and Illustration Software, 2025).

## Results

### Prevalence and serovars of *Salmonella* in egg samples

In this study, 260 egg samples were tested for the presence of *Salmonella*. The results revealed 24 positive *Salmonella* samples, resulting in an overall prevalence rate of 9% (24/260). Among the positive ones, *Salmonella* was detected in 5% of the eggshell samples (13/260) and in 3% of the egg content (8/260). Additionally, 1% of the samples (3/260) were positive for *Salmonella* in both eggshells and the content components (Table 1).

**Table 1:** Prevalence of *Salmonella* detected in chicken egg samples stratified by contamination site (eggshell, egg content, or both).

Detection site	Incidence of salmonella (%)
Egg content	8/260 (3%)
Eggshell	13/260 (5%)
Egg content and eggshell	3/260 (1%)
Total prevalence	24/260 (9%)

Serovar identification obtained by conventional slide agglutination was fully consistent with *in silico* serotyping results derived from whole-genome sequencing across all isolates (Table 2). The predominant identified serovar was *Salmonella Enteritidis*, accounting for 17% (4/24) of positive samples, followed by *Salmonella serovars Heidelberg*, *Bareilly*, *Cerro,* and *Typhimurium* at 13% each (3/24). Less frequent *Salmonella* serovars included *Minnesota*, and *Braenderup*, *Concord*, *Typhimurium*|, *Lago*s, *Wien,* and *Montevideo*, each detected once at 4% (1/24) of the positive samples (Figure 3). Notably, serovar *Infantis* was isolated from both the shell and contents of a single egg sample.

**Table 2:** A table summarizes the distribution percentages of *Salmonella* serovars in eggshells, egg contents, and both.

	Number of isolates		
Serovars	Eggshell	Egg content	Total
Enteritidis	4	0	4
Typhimurium	1	2	3
Heidelberg	1	2	3
Bareilly	2	1	3
Cerro	3	0	3
Braenderup	1	0	1
Concord	1	0	1
Infantis	1	1	2
Minnesota	1	0	1
Montevideo	1	0	1
*Typhimurium/Lagos*	0	1	1
Wien	1	0	1
Total	17	7	24

The serovar identification based on conventional slide agglutination and *in silico* serotyping derived from whole-genome sequencing.

#### Phenotypic characterization of antimicrobial resistance

Phenotypic antimicrobial susceptibility testing (AST) showed variability across *Salmonella* serovars (Figure 4). Cefoxitin (FOX), sulfisoxazole (FIS), and nalidixic acid (NAL) exhibited the highest resistance rates across multiple isolates. The most resistant isolate was *S. Montevideo* (155083_SE-7), exhibited resistance to 16 antimicrobials across *β*-lactams, cephalosporins, fluoroquinolones, tetracyclines, and sulfonamides. Additionally, high resistance levels were also observed in *S. Minnesota* (182773_SE-16; 11 antibiotics), *S. infantis* (193967_SE-17; 15 antibiotics), and *S. Heidelberg* (193981_SE-18; 14 antibiotics).

Resistance to the third-generation cephalosporins such as cefotaxime (FOT) and ceftazidime (TAZ), was prevalent among *S. Minnesota*, *S. Heidelberg*, *S. infantis*, and *S. Cerro*. Resistance to penicillins (AMP, SAM, AMC) was also observed in multiple isolates, especially in *S. Heidelberg* and *S. Minnesota*. Interestingly, all isolates remained susceptible to carbapenems [imipenem (IMI), doripenem (DORI), and meropenem (MERO)] and azithromycin (AZI). Aminoglycosides [amikacin (AMI), gentamicin (GEN), tobramycin (TOB)] showed high susceptibility across various serovars, except for *S. infantis* and *S. Heidelberg*, which demonstrated resistance to streptomycin (STR).

Ciprofloxacin (CIP) and levofloxacin (LEV) exhibited intermediate susceptibility in some isolates, whereas nalidixic acid (NAL) demonstrated elevated resistance levels. Resistance to sulfonamides [Sulfamethoxazole (SMX), Sulfisoxazole (FIS), Trimethoprim–sulfamethoxazole (SXT)] was observed, particularly in *S. Montevideo*, *S. Heidelberg*, and *S. infantis*. The susceptibility of colistin (COL) and polymyxin B (POL) were generally retained (i.e., intermediate susceptibility) among isolates, with only one resistance in serovar *Typhimurium* (112699_SE-3).

### Genotypic characterization by whole genome sequencing analysis

#### Antimicrobial resistance genes

The WGS analysis revealed diverse ARG profiles among *Salmonella* isolates representing different serotypes and multiple antibiotic classes (Figure 5). The predominant ARGs were associated with aminoglycoside resistance, with the *aac(6′)-Iaa* gene detected in nearly all isolates, including those belonging to *S. typhimurium*, *S. enteritidis*, *S. Bareilly*, *S. Heidelberg*, and *S. Cerro*. Additional aminoglycoside resistance genes included *aac(3)-IVa, ant(3″)-Ia, aph(3″)-Ib, aph(3′)-Ia, aph(4)-Ia*, and *aph(6)-Id*.

Among the MDR profiles, certain isolates, particularly *S. infantis* (193967_SE-17), harbored multiple ARGs across several antimicrobial classes, including *β*-lactamases (*bla_CMY-2_*)_,_ fluoroquinolones (*qnrS1*), sulfonamides (*sul1*), tetracyclines [*tet(A)*], trimethoprim (*dfrA14*), and phenicols (*floR_2*). Similarly, *S. Minnesota* (182773_SE-16) carried *bla_CMY-2_* and *qnrB19*.

β-lactamase resistance genes, particularly *bla_CMY-2_* and *bla*_*CTX-M-65*,_ were detected in *S. Minnesota*, *S. infantis*, *S. Heidelberg*, and *S. Concord*, indicating resistance to extended-spectrum β-lactam antibiotics. In contrast, fluoroquinolone resistance genes (*qnrB19 and qnrS1*) were limited to *S. Minnesota* and *S. infantis*. Sulfonamide resistance genes (*sul1 and sul2*), tetracycline resistance genes [*tet(A)*], and the trimethoprim resistance gene (*dfrA14*) were commonly identified among isolates of *S. Heidelberg*, *S. Minnesota*, *S. infantis*, *S. Cerro*, and *S. Concord*, whereas phenicol resistance (*floR_2*) was exclusively detected in *S. infantis* and *S. Concord.*

As illustrated in Figure 5, the distribution of these ARGs was consistent with the observed phenotypic resistance patterns, particularly for β-lactams and fluoroquinolones. *S. infantis* (193967_SE-17) demonstrated the greatest genetic resistance load among all serovars.

### Plasmid replicons typing

A total of 17 distinct plasmid replicon types were identified across the 23 genomes (Figure 6). The accuracy and reliability of the in-silico prediction of plasmid typing results were validated by comparing the results with known replicon types from previously published full plasmid sequences. All the *S. typhimurium* isolates consistently carried IncFIB(S)_1(FN432031) and IncFII(S)_1 (CP000858) plasmid type, with an additional Col440I_1 (CP023920.1) replicon identified in the *Typhimurium/Lagos* isolate. *S. enteritidis* isolates demonstrated a greater number of plasmid replicon types, harboring combinations of IncFIB(S)_1 (FN432031), IncFII(S)_1 (CP000858), and ColRNAI_1 (DQ298019). *S. Heidelberg* strains exhibited the highest plasmid variability, with certain isolates containing IncX1_1 (EU370913) plasmid, while other isolates had IncA/C2_1 (JN157804), IncI1_1_Alpha (AP005147), ColRNAI_1 (DQ298019), and ColpVC_1 (JX133088). Notably, *S. Minnesota* isolates harbored 5 plasmid types [IncA/C2_1 (JN157804), ColRNAI_1 (DQ298019), Col440II_1 (CP023921.1), Col440I_1 (CP023920.1), and Col8282_1 (DQ995352)], potentially indicating higher horizontal gene transfer activity. Other serovars, including *S. Braenderup*, *S. Bareilly*, *S. infantis*, *S. Concord*, and *S. Cerro*, had unique plasmid profiles, harboring IncX2_1 (JQ269335), ColE10_1 (X01654), and IncN_1 (AY046276) plasmid type.

### Plasmid-associated antimicrobial resistance genes

Plasmid replicon screening with PlasmidFinder identified five distinct types across seven isolates (Figure 7). The IncA/C2 replicon IncA/C2_1 was most prevalent, detected in three isolates including *S. Minnesota* and *S. Heidelberg*, while a second IncA/C2_1 variant was present in three isolates all belonging to serovar Cerro. IncN, IncI1, and Col440I occurred sporadically. Among antimicrobial resistance genes, *tet(A)* was the most common, followed by intermediate frequencies of *sul2*, *qnrS1*, and *blaCMY-2*; *qnrB19*, *dfrA14*, and the *aph* family had low prevalence.

### Virulence genes

The analysis of virulence genes across *Salmonella* isolates revealed a total of 134 detections, corresponding to 80 genes that were universally present across all the isolates, and 54 distinct virulence genes encompassing functions such as adhesion, invasion, and type III secretion. Several adhesion-related genes were prevalent in most isolates encodin fimbrial structures or surface adhesins, such as csg (curli), fim (Type 1 fimbriae), lpf (long polar fimbriae), and pef (plasmid-encoded fimbriae), which enable bacterial attachment to host tissues and biofilm formation. Invasion and intracellular survival effectors were supported by genes such as invA, spaO, prgH, pipB, sopA, sipA, and multiple sse/ssp., which were located on the type III secretion system (T3SS) within *Salmonella* pathogenicity islands (SPIs). In addition, the injecting effector proteins (e.g., sop, sip, ssp., sse, and ste) were found to allow host cells to manipulate host signaling and function to facilitate bacterial uptake. Notably, the cdtB (typhoid toxin component), sodCI (periplasmic superoxide dismutase), and rck (serum complement resistance) were also present in select strains. Iron acquisition system genes were also identified, such as ent (enterobactin), fep, irp, fyuA, and ybt gene clusters, ensuring that *Salmonella* can scavenge essential metals in the nutrient-limited host environment. Lastly, the spv virulence genes (spvR, spvC, and spvB) and pef operon genes (pefA–D), which encode fimbriae, were uniquely identified in *S. typhimurium* and *S. enteritidis*, highlighting their enhanced pathogenic potential (Figure 8) with additional detailed profiles provided in Supplementary Figure S1.

### Multilocus sequence typing phylogeny

Seven-locus MLST assigned the collection to multiple STs (*n* = 29), and the MLST phylogeny showed clear serovar-aligned clustering. ST11 (*S. enteritidis*), ST19 (*S. typhimurium*), and ST15 (*S. Heidelberg*) were predominant and formed tight, largely monophyletic clusters with short branches, indicating few allelic differences across the seven loci. Likewise, ST362 (*S. Bareilly*) and ST1593 (*S. Cerro*) clustered consistently with their serovar designations. In contrast, isolates belonging to ST533 (*S. Concord*), ST32 (*S. infantis*), ST316 (*S. Montevideo*), ST548 (*S. Minnesota*), ST6168 (*S. Wien*), and ST1561 (*S. typhimurium*/*S. Lagos*) appeared as singletons or small, more divergent lineages.

### Pan-genome structure

The pan-genome structure revealed four major gene categories (Figure 9). Core genes (45.3%) were present in all *Salmonella* serovars. Cloud genes (31.8%) were distributed across most serovars but absent in some isolates. Shell genes (20.2%) were identified in a moderate number of serovars, while soft core genes (2.7%) occurred in a small subset of isolates.

## Discussion

The findings of this study reveal the prevalence, serovar diversity, and genomic profiles of *Salmonella* in all sites of commercial chicken egg samples, including eggshell and egg content, sold in Riyadh, Saudi Arabia. *Salmonella* was detected in 9% of the 260 examined egg samples, more frequently observed on eggshells (5%) than within the egg contents (3%). This variation between the eggshells and egg contents is expected due to the direct exposure of the eggshells to fecal matter, bedding, and environmental dust within poultry houses, while the internal contamination of the egg contents occurred due to the vertical transmission or trans-shell penetration (Arnold et al., 2014). From a public health standpoint, eggshell contamination poses a considerable danger for indirect transmission via cross-contamination during food preparation, especially when raw eggs are managed in household or food service environments (Alsufyani et al., 2025). In contrast, the detection of *Salmonella* within egg contents, though less frequent, raises significant concern as it signifies internal contamination that cannot be remedied by surface washing or shell hygiene. Therefore, in this study, the isolates obtained from egg contents were primarily linked to clinically significant serovars and exhibited an elevated presence of antibiotic resistance determinants. This finding indicates a potentially increased risk of consumer exposure to more virulent and antimicrobial-resistant Salmonella strains.

The results of *Salmonella* prevalence underscore the necessity for dual risk mitigation strategies, encompassing stringent hygienic practices during egg handling to minimize shell-to-food cross-contamination, and enhanced control measures at the production level to avert internal egg contamination and restrict the spread of resistant strains (Alsufyani et al., 2025).

The global distribution of *Salmonella* serovars varies significantly between countries and across regions within the same country (Jamal et al., 2021; European Food Safety Authority and European Centre for Disease Prevention and Control, 2024; Wang et al., 2024). This variation is influenced by multiple factors such as host-pathogen transmission dynamics, agricultural practices, and food production and processing (Ferrari et al., 2019). For instance, *S. enteritidis* stands out as a major serotype linked to poultry and eggs, contributing to numerous foodborne outbreaks across Europe, including France, Spain, the Netherlands, and the United Kingdom during the years 2021 and 2022 (European Centre for Disease Prevention and Control, European Food Safety Authority, 2022). Nonetheless, the prevalence of other serovars differs geographically. For example, Australia and New Zealand reported elevated contamination levels of *S. typhimurium* and *S. infantis* in eggs and poultry products (Chousalkar et al., 2018).

Our investigation reflects this global diversity in which revealed that the predominant serotypes identified were *S. enteritidis* (17%) and *S. typhimurium* (13%), in line with previous reports from Riyadh city and global literature (Moussa et al., 2010). Both serovars were commonly linked to egg-associated outbreaks and harbor virulence genes that have been associated with various public health threats, including meningitis (Bahramianfard et al., 2021; ÓhAiseadha et al., 2010). Notably, *S. enteritidis* was detected in four samples from eggshells only. This observation is consistent with its established association with surface contamination in eggs and poultry products. However, according to the literature, it has been shown that *S. enteritidis* has the ability to inhabit and colonize the reproductive tissues of chicken, specifically in the ovaries and oviducts, enabling vertical transmission and persistence inside the egg (Foley et al., 2013). This underscores its relevance as a foodborne pathogen and the importance of monitoring both external and internal contamination routes when assessing egg safety.

Other serovars, including *S. Heidelberg*, *S. Bareilly*, and *S. Minnesota,* were also detected, which adds to the significance of this study. Both *S. Heidelberg* and *S. Bareilly* were rank among the leading cause of salmonellosis in the US and were linked to outbreaks reported in eggs and poultry (European Centre for Disease Prevention and Control, European Food Safety Authority, 2022; Bahramianfard et al., 2021; ÓhAiseadha et al., 2010; Foley et al., 2013; Chittick et al., 2006). The identification of less commonly reported serovars like *S. Braenderup*, *S. Concord*, and *S. Minnesota* indicates a wider range of *Salmonella* strains in poultry environments in Saudi Arabia which is remarkable (Bayu et al., 2013; Garcia et al., 2022). Of note, *S. Minnesota* is gaining attention for its multidrug resistance and potential to cause invasive illness (83–85.93). The observed variations in *Salmonella* strains could be a result of discrepancies in management practices, environmental conditions, or additional factors that support the persistence of various *Salmonella* serovars within egg production facilities (Park et al., 2022).

This diversity of *Salmonella* serovars highlights the necessity for risk-based surveillance strategies that prioritize serovars according to prevalence, their correlation with human disease, potential for outbreaks, virulence traits, and antimicrobial resistance profiles. Serovars like *S. enteritidis* and *S. typhimurium* require specific focus due to their persistent link to egg-related outbreaks, capacity to induce serious human infections, and established significance in both global and local epidemiological data (Moussa et al., 2010; Foley et al., 2013). The identification of serovars such as *S. infantis, S. Heidelberg, and S. Minnesota* raises further concerns, as these serovars have been increasingly associated with antibiotic resistance and the global spread of high-risk clones via poultry production systems (European Centre for Disease Prevention and Control, European Food Safety Authority, 2022; Bahramianfard et al., 2021; ÓhAiseadha et al., 2010; Foley et al., 2013; Chittick et al., 2006).

Virulence factors play a crucial role in the ability of *Salmonella* to survive in the host and cause disease. In this study, 137 virulence-related genes were identified across all isolates, with 80 of them consistently present in every serovar. *pefA*, *fyuA*, and *rck* were among the most notable ones that contribute to adhesion, iron acquisition, and immune evasion, which are key traits that enhance persistence and pathogenicity of *Salmonella* (Figure 8). The *pefA* gene, found in *S. typhimurium*, *Typhimurium/Lagos*, *S. enteritidis*, and S. *Concord*, encodes plasmid-encoded fimbriae and involved in intestinal adhesion and biofilm formation, potentially increasing the strains’ ability to colonize host tissues and survive in the environment (Weng et al., 2022). The *fyuA* gene, which enables bacteria to acquire iron in low-iron environments through the yersiniabactin system, was detected in *S. Minnesota*, *S. infantis*, S. *Heidelberg*, and S. *Braenderup*. The presence of this gene may contribute to increased virulence and a greater risk of invasive disease (Weng et al., 2022). The *rck* gene, associated with resistance to complement-mediated cell lysis, was found in *S. typhimurium*, *S. enteritidis*, and S. *Concord*, supporting these strains’ ability to evade host immune responses and potentially cause systemic infections (Silva et al., 2017).

Another concerning finding was the presence of the *cdtB* gene in *S. Minnesota* and S. *Montevideo*, which is a part of the cytolethal distending toxin (CDT-B). This gene interferes with host immune function and promotes deeper tissue invasion, increasing the likelihood of severe illness (Ho and Slauch, 2001). Its detection in *S. Minnesota*, a serovar already known for its multidrug resistance, raises significant public health concerns, as it suggests the strain may be both harder to treat and more capable of causing invasive disease.

Certain genes were identified in *S. typhimurium*, including the *Salmonella* virulence plasmid genes (e.g., *spvC* and *spvR*), fimbrial adhesion factors (e.g., *pefA–D*), and intestinal persistence gene (e.g., *ratB*), suggest a strong adaptation for systemic infection and intestinal persistence (Ho and Slauch, 2001; Castilla et al., 2006; Aljindan and Alkharsah, 2020; Cherubin et al., 1986). These virulence genes, particularly those associated with *Salmonella* virulence plasmids (e.g., *spvR*, *spvB*, *spvC*), were supported by the consistent detection of IncFIB(S)_1 and IncFII(S)_1 plasmids in all *S. typhimurium* and *S. enteritidis* isolates that are well known to carry the *spv* operon (Ho and Slauch, 2001; Castilla et al., 2006; Aljindan and Alkharsah, 2020; Cherubin et al., 1986). Moreover, smaller Col-type plasmids were also identified, notably Col440I and ColRNAI in *S. typhimurium* and *S. enteritidis* isolates, respectively. While these Col plasmids typically encode bacteriocins rather than direct resistance or virulence genes, their presence may enhance environmental survival and bacterial competitiveness (Silva et al., 2017). Together, the presence of virulence genes associated with IncF plasmids (i.e., *spv*) with Col plasmids underscores the genetic complexity, adaptability, and pathogenic potential of these *Salmonella* strains.

In contrast, *S. Heidelberg* carried specific genes, such as *grvA* (a stress response regulator) and *sspH1/sspH2* (ubiquitin ligase effectors), which may contribute to improved survival mechanisms in both host environments and food production settings, rendering these strains resilient and adaptive (Ho and Slauch, 2001). Meanwhile, *S. Cerro* and *S. Bareilly* demonstrated reduced virulence profiles, with a limited presence of *SPI*-1 and adhesion-related genes such as *pefA*, and *sopA*, indicating that these serovars may have the tendency toward environmental persistence rather than high pathogenicity (Srednik et al., 2023; Alvarez et al., 2023).

Overall, all the virulence factors emphasize the capacity of specific serovars to induce more severe or invasive infections and stress the necessity of integrating virulence profiling into genetic surveillance and public health risk evaluation.

In this study, *Salmonella* susceptibility results revealed notable resistance in the *β*-lactamase inhibitor class, particularly in two important combination antibiotics: ampicillin/sulbactam and amoxicillin/clavulanic acid, across six isolates. The same isolates that were resistant to ampicillin/sulbactam were also resistant to ampicillin alone. A previous study by Aljindan and Alkharsah (2020) reported antimicrobial susceptibility patterns in clinical *Salmonella* isolates from the Eastern Province of Saudi Arabia, showing that penicillin and penicillin combined with β-lactamase inhibitors, including ampicillin and ampicillin/sulbactam, still exhibited good activity against *Salmonella* over 8 years (2014–2018) (Aljindan and Alkharsah, 2020). The susceptibility pattern of our isolates to penicillin antibiotics is consistent with those findings.

Cephalosporins, also known as cephems, are widely used antibiotics for treating severe salmonellosis in humans (Cherubin et al., 1986). In this study, we tested representatives from the second generation (cefoxitin), third generation (cefotaxime, ceftazidime, ceftriaxone), and fourth generation (cefepime). All *Salmonella* isolates were susceptible to cefepime, the first cephalosporin of the fourth generation.

A high incidence of resistance was observed against third-generation cephalosporins, which raising substantial medical concern. Persistent resistance to cephalosporins over time suggests the presence of extended-spectrum β-lactamases (ESBLs), which frequently confer resistance to this antibiotic class (Shaikh et al., 2014). Fortunately, all *Salmonella* isolates in this study were screened for ESBL production, and only one isolate (*S. infantis*) tested positive. This isolate carried the IncFIB(K)_1_Kpn3 plasmid, which is strongly associated with the dissemination of *bla*_CTX-M-65_ in *S. infantis*. This genetic profile aligns with findings by Alzahrani *et.al*, who reported *S. infantis* isolates from chicken meat in Saudi Arabia harboring the same resistance genes and plasmid types, suggesting a potential link between contaminated poultry and the spread of MDR *S. infantis* (Alzahrani et al., 2023).

The repeated detection of the IncFIB(K)_1_Kpn3 plasmid in multiple studies underscores its role as a mobile genetic element that facilitates persistence and dissemination of resistance genes in *Salmonella*, particularly in *S. infantis* (Srednik et al., 2023). Globally, *S. infantis* has recently emerged as a major public health concern, particularly in Europe, England, and Wales, where strains carrying the megaplasmid pESI (plasmid of emerging *S. enterica Infantis*) have been reported. These plasmids enhance both multidrug resistance and virulence traits (Alvarez et al., 2023; Lee et al., 2021).

Maintaining the effectiveness of widely used third-generation cephalosporins, while ensuring the continued efficacy of fourth-generation agents such as cefepime, remains crucial in safeguarding treatment options against *Salmonella* (Wilson, 1998).

Quinolones and fluoroquinolones are considered as the first line treatment for adult worldwide. However, *Salmonella* isolates have been increasingly developing resistance to fluoroquinolones (CDC, 2024c). A study by Aljindan et al. (2018) reported a resistance prevalence of 19%, a high rise compared to 3% reported in 2013 (Aljindan and Alkharsah, 2020; Elhadi et al., 2013). Our findings, consistent with these and other studies, confirm the presence of fluoroquinolone-resistant *Salmonella* in Saudi Arabia (Aljindan and Alkharsah, 2020; Elhadi et al., 2013; Aljindan et al., 2018). In particular, we observed that the same isolates resistant to nalidixic acid also displayed intermediate to resistant levels against ciprofloxacin and levofloxacin. This resistance is a significant public health concern, especially if such strains cause salmonellosis in children, adults with invasive infections, or immunocompromised individuals (CDC, 2025).

The CDC has long recognized *Salmonella* resistance to conventional antimicrobial drugs such as ampicillin, chloramphenicol, and trimethoprim-sulfamethoxazole. None of these agents should be considered first-line treatments, which is consistent with our findings, as we also observed resistance to these antibiotics in our isolates (CDC, 2024c). Another notable MDR serovar identified in this study was *S. enteritidis*. As previously noted, *S. enteritidis* is the most common serovar associated with human infections in Riyadh, Saudi Arabia (Alghoribi et al., 2019).

*Salmonella* Minnesota was detected once in this study, and it was MDR. In 2021, Saudi Arabia reported that a strain of *S. Minnesota* isolated from chicken carried the mcr-9 gene, which confers colistin resistance (Alzahrani et al., 2021). In that report, the strain was phenotypically sensitive to colistin but resistant to amoxicillin-clavulanic acid, ampicillin, sulfisoxazole, and tetracycline. Our results for the two *S. Minnesota* isolates were consistent with those findings, except that colistin resistance was interpreted as intermediate, following the Clinical and Laboratory Standards Institute (CLSI) 2024 M100, 33th edition, which updated the MIC breakpoints for colistin from “sensitive” to “intermediate” (Alzahrani et al., 2021; Clinical and Laboratory Standards Institute, 2024). Additionally, *S. Minnesota* isolates from Saudi poultry have been shown to carry the *cdtB* toxin gene, which induces DNA damage and cell cycle arrest, potentially leading to typhoid-like disease in humans, in agreement with findings that one of the two isolates *S. Minnesota* was harboring the same gene (Alzahrani et al., 2023).

Recent genomic surveillance in Riyadh detected two S. Minnesota ST548 isolates from retail chicken meat that showed phenotypic colistin resistance and harbored the plasmid-borne mcr-1.1 gene, alongside multiple resistance genes (*sul2, tetA, blaTEM-1B, qnrB19, aac(6′)-Iaa, floR*) and various plasmid replicons (Mukhtar et al., 2022). These data collectively indicate that Saudi S. Minnesota strains possess a broad resistome and plasmids that support horizontal gene transfer. In our analysis, S. Minnesota isolate demonstrated multidrug resistance profiles and possessed the cdtB virulence gene, confirming existing literature and highlighting concerns that this serovar may combine heightened virulence with the ability to acquire and transmit clinically significant resistance genes. Collectively, our observations emphasize S. Minnesota as a serovar requiring prioritized focus in national AMR surveillance and poultry food safety initiatives. In 2023, *S. Minnesota* and *S. Heidelberg* were investigated in Brazil, where isolates from poultry and other sources were compared with international strains (Rodrigues et al., 2020). The study confirmed that both serovars are highly infectious and MDR, posing significant public health concerns worldwide (Rodrigues et al., 2020).

In our study, *S. Montevideo* was also identified and found to harbor the *cdtB* toxin gene. This serovar can infect chickens, often without causing noticeable symptoms (Lalsiamthara and Lee, 2017). Infected birds may carry *S. Montevideo* asymptomatically, creating a transmission risk through direct contact or consumption of contaminated poultry products, including eggs (Lalsiamthara and Lee, 2017). Notably, the *S. Montevideo* isolate in our findings was detected in the egg content, suggesting systemic infection in the hen.

The antimicrobial resistance gene profiles determined in this study align with widely documented patterns in poultry-associated *Salmonella*. The prevalent identification of aminoglycoside resistance genes, especially *aac*(6′)-*Iaa* among various serovars, indicates a frequently documented intrinsic resistance characteristic in *Salmonella* isolates globally (Abdelhamid and Yousef, 2023). Comparable distributions of aminoglycoside-modifying enzymes, including *aph* and *ant* gene families, have been consistently reported in poultry-derived *Salmonella* from Europe, Asia, and North America (Zhang et al., 2023).

The detection of plasmid-mediated *β*-lactamase genes, specifically *bla*_CMY-2_ and *bla*_CTX-M-65_, in serovars such as *S. infantis*, *S. Minnesota*, *S. Heidelberg*, and *S. Concord* corresponds with global accounts of the widespread occurrence of *Amp*C and extended-spectrum β-lactamases in foodborne *Salmonella*. These resistance determinants are frequently linked to multidrug-resistant clones prevalent in chicken production systems (Aravena et al., 2024).

The identification of plasmid-mediated fluoroquinolone resistance genes (*qnrB19 and qnrS1*), alongside sulfonamide (*sul1, sul2*), tetracycline [*tet(A)*], and trimethoprim (*dfrA14*) resistance genes, reflects resistance patterns often documented in poultry-associated *Salmonella* worldwide (Aravena et al., 2024). The significant genetic resistance load identified in *S. infantis* in this investigation aligns with its designation as a globally distributed high-risk serovar linked to antibiotic resistance and intensive chicken farming (Alvarez et al., 2023).

These findings situate the identified resistance gene patterns within a global framework and emphasize the role of poultry-associated *Salmonella* in Saudi Arabia in the global reservoir of antimicrobial resistance determinants, highlighting the necessity of incorporating whole-genome sequencing into national and international surveillance systems.

In this study, we also investigated the association between ARGs and plasmids among the *Salmonella* isolates (Figure 7). It was found that certain ARGs were primarily linked to specific plasmid types, suggesting possible horizontal gene transfer (Aviv et al., 2016). Interestingly, we found no clear correlation between the IncFIB(K)_1_Kpn3 plasmid and ARGs, despite the *S. infantis* isolate carrying about nine resistance genes. This discrepancy could be explained by mutations or partial deletions in the plasmid that disrupted its expected association with ARGs (Aviv et al., 2016). Alternatively, the resistance genes may have been integrated into the bacterial chromosome, making them independent of plasmid carriage (García-Soto et al., 2020).

Moreover, the IncFIB(K)_1_Kpn3 plasmid was associated with virulence factors. Although ARGs were not directly linked to this plasmid, it carried virulence determinants, consistent with reports that such plasmids can facilitate the horizontal transfer of virulence factors (Hull et al., 2022). The presence of virulence genes, even in the absence of resistance determinants, suggests that IncFIB(K)_1_Kpn3 enhances pathogen fitness by promoting adhesion, invasion, or immune evasion (García-Soto et al., 2020). These factors may indirectly support antimicrobial resistance by improving bacterial survival and persistence in hosts (García-Soto et al., 2020). Further investigations, including transcriptome analysis, plasmid profiling, and functional analysis of bacterial pathogenicity, are necessary to understand the functional significance of this plasmid. Especially, growing evidence indicates that large plasmids in *S. infantis* can acquire extra resistance genes and expand into megaplasmids, thereby increasing bacterial survival and accelerating the dissemination of AMR (García-Soto et al., 2020).

In this study, we also observed that the only isolate resistant to colistin and polymyxin B lacked the recognized genes typically associated with resistance (such as *mcr* genes, *pmrA, pmrB, phoPQ,* and *mgrB*) (Moghadam et al., 2022; Leite et al., 2020). In addition, the absence of the plasmids IncI2, IncX4, and IncHI2, which are the most frequently reported carriers of *mcr* genes, suggests a potential alternative resistance mechanism (Moghadam et al., 2022). Interestingly, the presence of IncFII(S)_1 plasmid indicates that plasmid-associated regulatory elements may contribute to this resistance, as it is reported that its backbones are involved in the dissemination of colistin resistance, as they are known to commonly carry *mcr* genes and other resistance determinants (Zelendova et al., 2021). Although no *mcr* genes were detected, the isolate’s resistance to colistin, along with the carriage of this plasmid and the *aac*(6′)-*Iaa* gene (which encodes resistance to aminoglycoside antibiotics), raises major concern. This genetic configuration could facilitate the horizontal transmission of antimicrobial resistance along the food chain.

The serovar *S. Heidelberg* was isolated three times, and according to the phylogenetic tree (Figure 2), the isolates clustered within the same branch. Among them, two closely related isolates harbored the *bla*_CMY_ gene. The presence of *bla*_CMY_ on IncX1 plasmids is particularly important, as these plasmids are recognized as key vectors for the transmission of *AmpC*-type *β*-lactamase resistance genes, conferring resistance to multiple β-lactam antibiotics, including third-generation cephalosporins (Dobiasova and Dolejska, 2016). The identification of these resistance determinants on IncX1 plasmids underscores their ability to mediate rapid horizontal gene transfer among bacterial populations. This highlights their role in the dissemination of multidrug-resistant organisms from food sources to humans, representing a significant public health concern.

Pan-genome analysis enhances outbreak investigations and molecular epidemiological studies by facilitating high-resolution comparisons of gene content among isolates (Barh et al., 2020). In addition, it offers a structure for understanding the genetic diversity and epidemiological patterns of *Salmonella*, beyond single-reference genome methodologies (Barh et al., 2020; Muigano and Musila, 2025). The pangenome structure of all *Salmonella* in this study demonstrated a majority of core genes (45.3%), conserved across all serovars and critical for *Salmonella* survival. The conserved genes confirm their significance for species identification and molecular diagnostic applications, ensuring precise and consistent detection across several serovars (Barh et al., 2020). The significant percentage of auxiliary genes, comprising cloud genes (31.8%) and shell genes (20.2%), underscores notable genomic heterogeneity among isolates. This variable gene pool is significant because it often contains factors linked to antimicrobial resistance, virulence, and environmental adaptation, elucidating the strain-specific variations in pathogenic potential and resistance profiles identified in this study. These findings collectively illustrate that incorporating pan-genome analysis into *Salmonella* surveillance enhances diagnosis, improves predictions of resistance and virulence, and offers a transferable and scalable framework applicable to food safety and public health monitoring programs in Saudi Arabia and other regions.

The findings of this study have significant implications within a One Health framework, highlighting the interconnected roles of environmental, agricultural, and human health sectors in the epidemiology of *Salmonella* and antibiotic resistance. The identification of many *Salmonella* serovars and resistance factors in chicken eggs underscores the complex nature of contamination routes throughout the food production chain and emphasizes the necessity for comprehensive surveillance strategies.

Furthermore, the findings underscore the importance of risk-based monitoring and management strategies in egg production and distribution from an agricultural and food safety standpoint. The genomic characterization of *Salmonella* serovars and their antibiotic resistance profiles yields data that can facilitate targeted surveillance techniques and improve the early detection of serovars pertinent to public health.

The existence of clinically significant and antimicrobial-resistant *Salmonella* serovars in eggs emphasizes the importance of integrated surveillance between food and clinical sectors in accordance with human health. The utilization of whole-genome sequencing for foodborne pathogens provides a significant resource for enhancing risk assessment, promoting antimicrobial stewardship, and improving collaboration within environmental, agricultural, and public health domains. Therefore, integrating genetic data from foodborne isolates with human health surveillance enhances source attribution and fortifies epidemic investigation capabilities. In Saudi Arabia, these results correspond with government initiatives to improve food safety regulations and monitor antibiotic resistance through a One Health framework.

While this study provides the first in-depth genomic characterization of *Salmonella* in chicken eggs in Saudi Arabia, several limitations should be acknowledged, the sampling was limited to chicken eggs from selected regions, which may not fully represent the national distribution of *Salmonella*; thus, expanding the sampling across other regions and times would enhance future surveillance efforts. In addition, the cross-sectional approach offers essentially a snapshot of contamination and antimicrobial resistance patterns, highlighting the necessity for long-term monitoring to observe developing clones and resistance trends.

Lastly, it would be valuable to expand future research under a One Health framework, including animal, food, environmental, and human *Salmonella* isolates. This would offer a more thorough comprehension of the transmission dynamics of *Salmonella* and associated antimicrobial resistance factors throughout the food chain and public health domains.

## Conclusion

This study provides the first of its kind a detailed genomic analysis of *Salmonella* contamination in chicken eggs in Saudi Arabia by combining whole-genome sequencing with phenotypic antimicrobial susceptibility testing. The paper provides a comprehensive analysis of serovar diversity, antimicrobial resistance, and virulence profiles linked to egg production. The identification of epidemiologically significant serovars, including *S. enteritidis* and *S. typhimurium*, as well as emerging serovars such as *S. Minnesota*, *S. Heidelberg*, and *S. Montevideo*, which possess clinically pertinent resistance determinants, emphasizes the potential public health hazards linked to poultry eggs and accentuates the increasing apprehension regarding antimicrobial resistance in poultry settings.

These findings underscore the necessity for enhanced, risk-based surveillance of *Salmonella* in egg production systems, focusing specifically on serovars of public health significance and those exhibiting multidrug resistance and virulence factors. Incorporating genetic surveillance into standard monitoring methods can facilitate the early identification of high-risk clones, refine source attribution, and guide targeted actions throughout production, processing, and distribution phases. Moreover, strengthening antimicrobial stewardship procedures in chicken production is crucial to curtail the selection and spread of resistant strains across the food chain.

Considering the evolving nature of antimicrobial resistance, future study needs to concentrate on continuous and multi-regional investigations to observe dynamic trends in serovar distribution, resistance patterns, and virulence profiles. These approaches will provide essential insights into the development and existence of antimicrobial-resistant *Salmonella* in Saudi poultry systems, thereby enhancing national risk assessment, One Health surveillance, and evidence-based policymaking to mitigate foodborne salmonellosis and antimicrobial resistance.

## Data Availability

The datasets presented in this study can be found in NCBI GenBank under BioProjects PRJNA1096000.

## Footnotes

1
https://doi.org/10.12688/f1000research.15931.2

2
https://doi.org/10.1093/bioinformatics/btu170

3
https://doi.org/10.1089/cmb.2012.0021

4
https://doi.org/10.1093/bioinformatics/btt086

5
https://doi.org/10.12688/wellcomeopenres.14826.1

6
https://doi.org/10.1099/mgen.0.000748

7
https://doi.org/10.1128/AAC.02412-14

8
https://doi.org/10.1093/nar/gki008

## Supplementary material

Supplementary Figure S1 Heatmap of gene presence/absence where rows are isolates (labeled by Sample ID and serovar) and columns are individual genes grouped by functional category. Category headers denote the molecular function/type (e.g., Adhesins/Outer membrane, F4 fimbriae, long polar fimbriae, ixidative stress, plasmid-encoded fimbriae, regulator, resistance to killing, siderophore systems, SPI-1 apparatus, SPI-1 effectors, SPI-2 effectors, toxin, type 1 fimbriae, virulence plasmid). The color scale is binary: white/light = 0 (absent) and dark blue = 1 (present). SPI-1/2 refer to Salmonella Pathogenicity Islands 1 and 2.
