# Supplementary figures and images for: Genomic insights into the prevalence and genetic diversity of Salmonella in chicken eggs in Saudi Arabia
Source: Front Microbiol. 2026 Feb 25;17:1760213. doi: 10.3389/fmicb.2026.1760213 (PMC12975983; doi:10.3389/fmicb.2026.1760213)

## Virulence Factors Presence/Absence

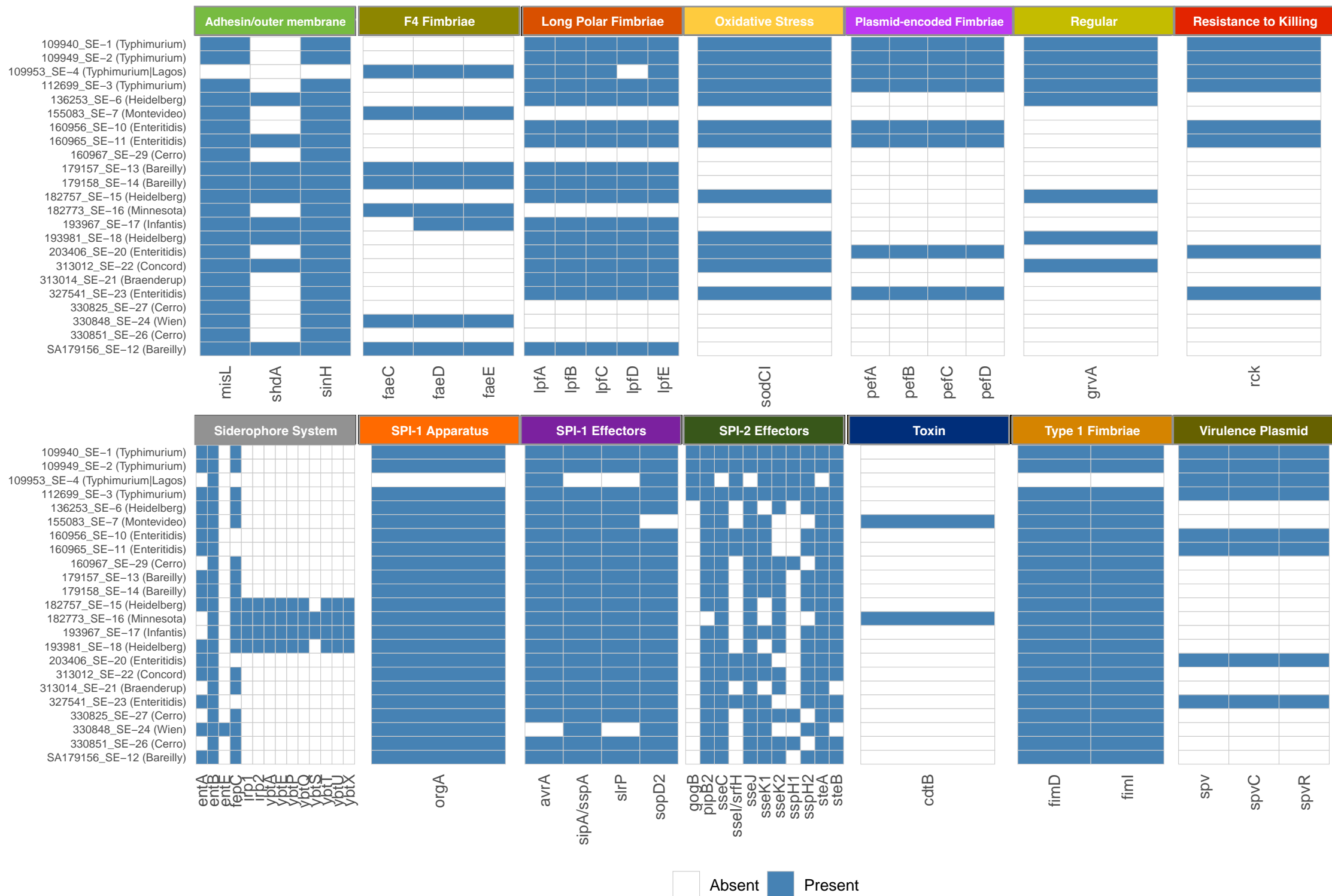

Supplement: Supplementary file 1 [file Data_Sheet_1.pdf]
